# Effect of metformin in a novel experimental model of peripheral artery disease

**DOI:** 10.1042/CS20243343

**Published:** 2025-08-13

**Authors:** Smriti Murali Krishna, Joseph Moxon, Ann-Katrin Kraeuter, Jonathan Golledge

**Affiliations:** 1 The Vascular Biology Unit, Queensland Research Centre for Peripheral Vascular Disease, College of Medicine and Dentistry, James Cook University, Townsville, Queensland 4811, Australia; 2 The Australian Institute of Tropical Health and Medicine, Townsville, Queensland, Australia; 3 School of Molecular Science, La Trobe University, Kingsbury Drive, Bundoora, Victoria, Australia; 4 Faculty of Health and Life Sciences, Psychology, Northumbria University, Newcastle upon Tyne, U.K; 5 Brain Performance and Nutrition Research Centre, Northumbria University, Newcastle upon Tyne, U.K; 6 NUTRAN, Northumbria University, Newcastle upon Tyne, U.K; 7 Department of Vascular and Endovascular Surgery, Townsville University Hospital, Townsville, Queensland, Australia

**Keywords:** ischaemia, metformin, peripheral arterial disease, preclinical models

## Abstract

Limited drug therapies for peripheral artery disease (PAD)-related walking impairment exist. There has been a recent interest in repurposing the diabetes medication metformin to treat PAD. Animal studies designed to develop new PAD drug therapies have mainly used a model of temporary hind limb ischaemia (HLI). The aim of this study was to test whether metformin improved blood supply and ambulation in a novel mouse model with ongoing HLI. Stable HLI was created in apolipoprotein E-deficient mice by a two-stage surgical procedure. Five days after HLI was induced, mice were randomly allocated to receive metformin (*n* = 16; 300 mg/kg/day) or vehicle control (*n* = 15) by oral gavage for four weeks. The primary outcome was hind limb blood supply assessed by laser Doppler. Other outcomes included treadmill performance and molecular changes in the ischaemic limb. Metformin improved hind limb blood supply (*P*<0.001), but not physical performance, associated with increased phosphorylation of 5′ adenosine monophosphate-activated protein kinase and endothelial nitric oxide synthase (*P*<0.05), reduced expression of thioredoxin interacting protein (*P*<0.05) and increased expression of peroxisome proliferator-activated receptor gamma coactivator 1-alpha (*P*<0.05) in the ischaemic muscles and increased circulating nitric oxide levels (P<0.05). Metformin improved blood supply in a novel model of limb ischaemia associated with molecular changes previously linked with promoting angiogenesis, but these changes did not translate to improved physical performance. The findings suggest that laser Doppler hind limb blood supply may not be an ideal outcome measure to gauge the success of a drug in patients with PAD-related walking impairment.

## Introduction

Peripheral arterial disease (PAD) affects about 6% of adults and causes impaired walking and reduced quality of life [1,2]. The main treatments for symptomatic PAD are exercise programmes and minimally invasive or open surgical operations to restore blood supply to the leg, but these therapies have substantial limitations [3,4]. One medication, cilostazol, is available for the leg symptoms of PAD but is not recommended by some current practice guidelines [5,6]. There is a need for effective drugs to treat the poor blood supply, impaired physical performance and pain experienced by people with PAD.

Rodent models are commonly used to discover and test drug therapies for cardiovascular diseases, but there have been some translational challenges [7,8]. PAD is most commonly modelled in young rodents by ligating or excising the femoral artery, which produces acute hind limb ischaemia (HLI), but this fails to simulate the usual clinical presentation of chronic limb ischaemia, with mice having no impairment in ambulation on a treadmill typically found in patients with PAD [8,9]. In addition, limb blood supply in the traditional HLI model spontaneously recovers, making it impractical to test the effect of drugs on the outcome of established stable limb ischaemia [9,10]. Others have reported using ameroid constrictors to progressively occlude the femoral artery over two weeks resulting in stable but mild limb ischaemia in rodents [11]. We recently developed a two-stage model that involved placing two ameroid constrictors on the femoral artery for two weeks to promote slow occlusion, followed by excision of the femoral artery segment and collaterals in between the two ameroid constrictors [9]. The two-stage model induces severe and stable HLI resulting in impaired physical performance, which is improved by exercise training as seen in patients with PAD [9]. The two-stage model, therefore, has a greater potential to test PAD drug therapies than the traditional femoral artery ligation approach, which mimics transient ischaemia.

Metformin is a member of the biguanide drug class commonly used to treat type 2 diabetes [12]. Metformin acts on mitochondrial function to increase the adenosine monophosphate to adenosine triphosphate ratio, thereby indirectly activating 5′ adenosine monophosphate-activated protein kinase (AMPK) [12]. AMPK activation has been proposed as a therapeutic target to treat ischaemia in a range of organs and tissues, including the muscles of the limb [13,14]. AMPK deficiency has been reported to impair recovery from acute HLI in mice [15,16]. Conversely, administering metformin to rodents following acute HLI induction is associated with improved blood supply to the ischaemic limb because of AMPK activation which in turn stimulates endothelial nitric oxide synthase (eNOS), thereby increasing nitric oxide (NO) and promoting angiogenesis [17]. Metformin has also been shown to interact with other targets such as thioredoxin interacting protein (TXNIP) [18] and peroxisome proliferator-activated receptor gamma coactivator 1-α (PGC1α) [19,20], which could play a role in promoting angiogenesis. This, in addition to evidence from some small clinical trials, suggests that metformin may have value in improving blood supply in patients with PAD; however, the clinical relevance of findings in the traditional HLI rodent model remains unclear [21,22]. Several current clinical trials aim to test whether metformin is an effective drug therapy for PAD-related walking impairment; however, there is a need to further evaluate the mechanisms by which metformin improves limb perfusion and examine its effects on ambulation in a clinically relevant model [23]. The aim of this study was to assess whether the administration of metformin improved blood supply and ambulation in a treadmill test within a novel mouse model of stable HLI [9].

## Materials and methods

### Study design, group allocation and protocol

**Figure 1:**
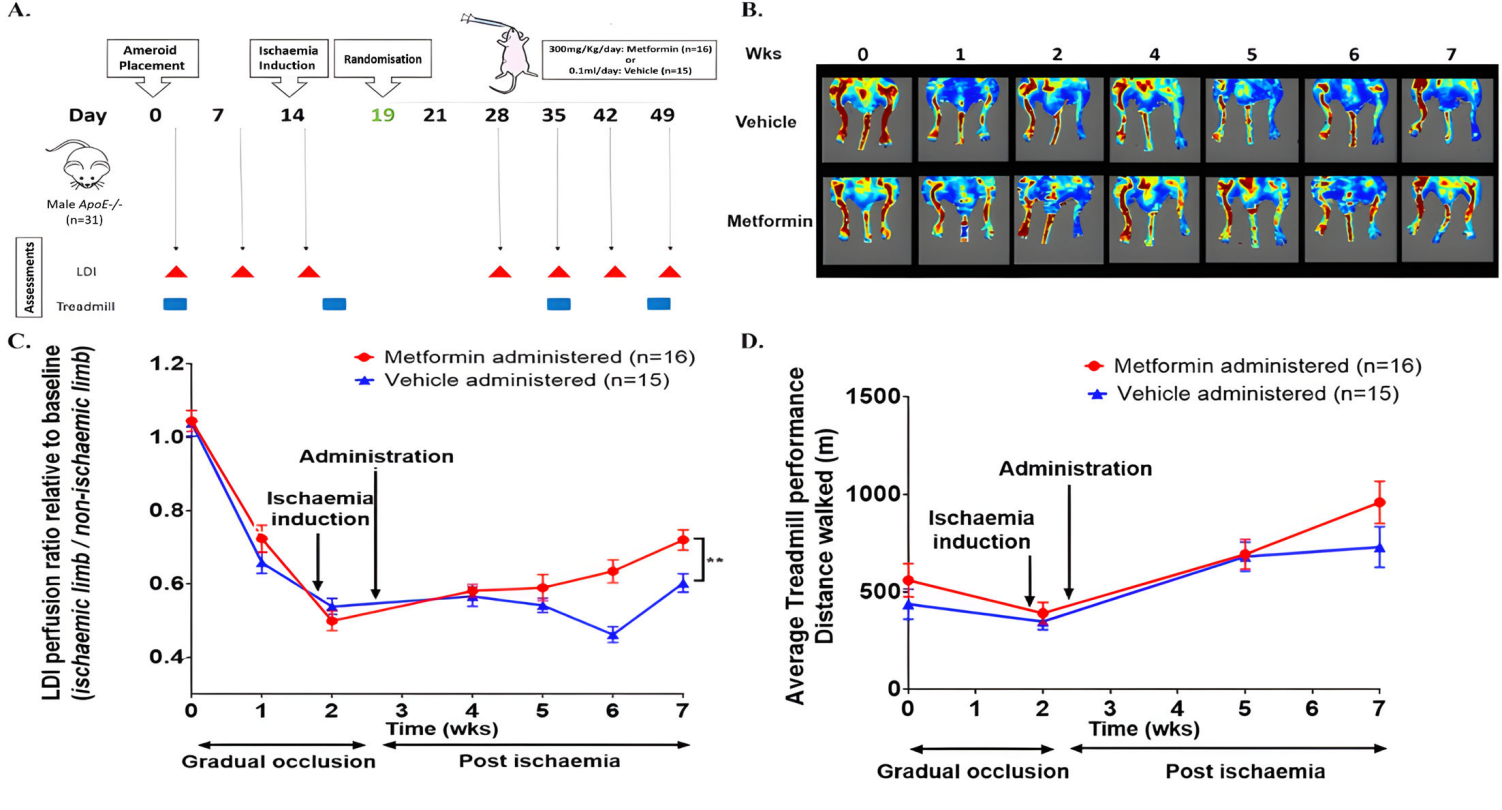
Effect of metformin administration on hind limb blood supply in the two-stage mouse model of PAD. **(A**) Study design for examining the effect of metformin on hind limb ischaemia (HLI) in apolipoprotein E-deficient (*ApoE^-/-^*) mice. Mice were randomised five days after ischaemia induction and subjected on daily intervention with metformin or vehicle. Laser Doppler imaging (LDI) assessments were carried out at baseline, days 7, 16, 28, 35, 42 and 49. Treadmill walking distance tests were performed at baseline, days 16, 35 and 45. (**B**) Representative LDIs at the assessment times of the intervention groups. (**C**) LDI limb perfusion ratios in *ApoE^-/-^* mice receiving metformin (*n* = 16) and vehicle control (*n* = 15). Values are normalised to the means of the baseline for each group, and data are expressed as mean ± SEM. Linear mixed effect (LME) analysis suggested a significant difference (*P<*0.001) between groups over the experimental period. (**D**) The effect of metformin on treadmill exercise performance in the two-stage mouse model of PAD. Treadmill distance travelled by metformin-administered (*n* = 16) and vehicle-administered groups (*n* = 15). Administration commenced five days post ischaemia. Data expressed as mean ± SEM. Data were analysed by LME and square root transformed to fit model assumptions. The treadmill distance travelled showed no significant difference over time (*P=*0.241) in mice receiving metformin and vehicle. PAD, peripheral artery disease.

Mice were housed in a closed tecniplast system (Buguggiate, Italy) with environmental enrichment at 22±1°C with a 12-h dark/light cycle and *ad libitum* access to water and standard diet (Goldmix Stockfeeds, Norco, Lismore, NSW, Australia). The study was designed as a randomised controlled trial [9]. HLI was created in 31 six-month-old male apolipoprotein E-deficient (*ApoE^-/-^*) mice (*n* = 31) by a two-stage surgical procedure [9], and after five days, mice were randomised using a random number generator to receive metformin (*n* = 16; 300 mg/kg/day) or vehicle control (*n* = 15; distilled water) (Figure 1A) by oral gavage. Mice of only one sex were used as a previous study indicated that there were sex differences in muscle function following HLI [24]. Six-month-old *ApoE^-/-^* mice were used as these animals have atherosclerosis and dyslipidaemia as typical found in patients with PAD. The dose of metformin chosen was estimated to equate to a daily dose of approximately 1500 mg in humans and has been previously reported to achieve therapeutic plasma levels in mice [25,26]. Drug or vehicle control was administered to mice by an independent research assistant via gavage, so that outcome assessors and investigators remained blinded to the group allocation. Drug administration continued for four weeks after which mice were killed by CO_2_ asphyxiation, and ischaemic gastrocnemius muscle tissue samples were harvested into optimal cutting compound (OCT, ProSciTech) or in RNA Later (Qiagen). OCT-embedded samples were snap-frozen in liquid nitrogen and stored in −80°C, and RNA Later samples were stored at −20°C for later analysis. Outcome measures included hind limb blood supply, physical performance and molecular pathways implicated in the control of angiogenesis.

### HLI model

Unilateral left HLI was created in *ApoE^-/-^* mice (*n* = 31) through a previously described two-stage operation [9]. The left femoral artery was exposed, and two custom-made ameroid constrictors of 0.25 mm internal diameter (Research Instruments SW) were placed around it, one immediately distal to the inguinal ligament and one proximal to the sapheno-popliteal bifurcation. After 14 days, a new incision was made, and the ligated femoral artery segment between the two ameroid constrictors and collaterals was excised.

### Laser Doppler imaging

The primary outcome measure was limb blood supply measured by laser Doppler imaging (LDI, moorLDI2, Moor Instruments) performed at baseline (before first surgery), two days after ischaemia induction and two and four weeks after commencing metformin or vehicle administration (Figure 1A), according to a previously published protocol [9]. Hair was removed by epilating cream from both limbs on the day prior to LDI. Images of the left ischaemic and contralateral right non-ischaemic limb were acquired under the same experimental conditions. Ambient light and temperature (maintained at 37°C core temperature) were carefully controlled to minimise background variations. Mice were placed in dorsal decubitus and anaesthetised using isoflurane (2.0% induction dose and 1.8% maintenance dose plus oxygen; 1 L/min, VET Equip). The LDI system was mounted on a movable track that was fixed 26 cm above the limbs, while the animals were restrained on a warming table with a black surface. Image analysis software (Laser Doppler Perfusion Measure, V3.08, Moor Instruments) was used to calculate mean flux units, which represented a quantitative analysis of tissue perfusion on a scale of 0 to 1000 perfusion units (PU). Limb perfusion was expressed as the ratio of the flux value of the ischaemic limb relative to the value of the contralateral non-ischaemic limb in the same mouse and presented as average PU.

### Physical performance assessment

A treadmill test was performed at baseline (before first surgery), two days after ischaemia induction and two and four weeks after commencing metformin or vehicle administration (Figure 1A), as previously described to determine ambulatory distance [9]. A six-lane Excer3/6 Treadmill (Columbus Instruments) was used. The treadmill belt and lanes were cleaned with water and 70% alcohol and dried with paper towel before each test to remove any body scent. Mice were acclimatised to the treadmill by ambulating at 5 m/min for 5 min once daily on three consecutive days prior to starting testing. Before each treadmill test, mice were fasted for 1 h. The speed of the treadmill was controlled using the built-in software and calibrated using an inbuilt speedometer mounted on the treadmill platform. A treadmill test involved an initial warm up at 5 m/min for 5 min followed by a progressive speed increase from 5 to 10 m/min, accelerated at 1 m/min. Following this, the treadmill speed remained at 10 m/min for up to a total running time of 20 min. No incline was used. During the test, a stimulus grid of 3 Hz was kept on until mouse exhaustion, defined as ten returns to the stimulus grid as previously reported [9]. The treadmill software recorded the total distance walked by a mouse until exhaustion. An observer blinded to the group allocation supervised the experiment.

### Plasma analysis

At the end of the experiment, blood was collected by cardiac puncture into heparin-coated tubes (BD Microtainer). Platelet poor plasma was generated by centrifuging blood at 4000×g at 4°C for 10 min, and supernatants were centrifuged for an additional 10 min (10,000×g) at 4°C. Plasma samples (*n* = 9/group) were randomly selected and processed for NO assessment. Plasma NO levels were estimated by measuring the concentration of the stable end products nitrate and nitrite using a commercial kit (Cayman) based on the Griess reaction following the manufacturer’s protocol. The absorbance was measured at 540 nm (Polarstar Omega).

### Western blotting

Protein was extracted from the randomly selected gastrocnemius muscles samples harvested from the ischaemic limbs of the mice randomly selected from both study groups (*n* = 7–8/group). Tissues were thawed on ice, rinsed in PBS and transferred to 500 µl of ice-cold radio immunoprecipitation assay buffer (Cell Signalling Technology) containing protease inhibitors (Roche) and phosphatase inhibitors (PhosSTOP). Tissues were homogenised and centrifuged; the supernatants were collected; and protein concentrations were quantified by the BioRad protein assay (BioRad, U.S.A.). Protein samples (15 µg) were mixed with Laemmli buffer containing dithiothreitol (DTT; 0.39 mg per 1 ml of Laemmli buffer; Bio-Rad, U.S.A.) and denatured and loaded into the SDS-polyacrylamide electrophoresis pre-cast gels (4–15%, BioRad), along with Precision Plus Protein™ WesternC™ Protein Standard (BioRad). Following the separation of the proteins by electrophoresis, the proteins were transferred to polyvinylidene fluoride (PVDF) (Biorad or Immobilin FL, Licor). PVDF membranes were Ponceau stained to confirm successful transfer and blocked with 5% ECL prime blocking agent and then incubated with primary antibody overnight at 4°C on a low-speed shaker. These included mouse monoclonal antibodies directed against AMPKα, phospho-AMPKα, total e-NOS, phospho-eNOS (Ser^1177^), glyceraldehyde 3-phosphate dehydrogenase (GAPDH), TXNIP or PGC1α. After washing, membranes were incubated in HRP-conjugated secondary antibody (goat anti-rabbit; 1:1000) (DakoCytomation, Denmark) at room temperature on the low-speed shaker for 2 hours. Membranes were washed in TBS-T and imaged using a LiCor Odyssey scanner, and band intensities were quantified using the Odyssey software. Protein expression of phospho-AMPKα and phospho-eNOS (Ser1177) was measured relative to total-AMPK and total-eNOS expression, respectively. GAPDH protein expression was measured to ensure equal protein loading between samples. The TIXNIP and PGC-A proteins were assessed by relative densitometry analysis of the respective protein levels relative to total protein expression. Antibodies and dilutions used are shown in Supplementary Table S1.

### Quantitative real-time polymerase chain reaction assays

Gastrocnemius muscles of ischaemic and non-ischaemic limbs of mice were harvested and immersed in RNA later (Qiagen), placed on ice and subsequently stored at −20°C. Total RNA was isolated from randomly selected ischaemic gastrocnemius muscle samples (*n* = 8/group) using the RNeasy Mini kit (Qiagen) according to manufacturer’s instructions. The quantitative real-time PCR (qRT-PCR) reactions were performed using QuantiTect SYBR Green one-step RT-PCR assay (Qiagen). For each gene of interest, the relative expression in each sample was determined by using the concentration-Ct-standard curve method and normalised to the expression of GAPDH. All samples were tested in duplicate. Qiagen QuantiTect Primer Assays (Qiagen) were used to assess the mRNA expressions of AMP-activated protein kinase (*Ampkα1)*, peroxisome proliferator-activated receptor gamma coactivator 1*α* (*Pgc1α*, thioredoxin-interacting protein (*Txnip)*, endothelial nitric oxide synthase 3 (*Nos3*) and glyceraldehyde 3-phosphate dehydrogenase (*Gapdh*) (Supplementary Table S2).

### Statistical analysis

Sample size was estimated to test the primary hypothesis that metformin administration for four weeks improved blood supply by 30% within the novel two-stage HLI model. To calculate the expected outcomes, LDI results from a previous study using the acute HLI model were used [17]. In that study, ten mice were imaged for 28 days after ischaemia induction and mean (standard deviation, SD) flux ratio of the control group was 0.51 (0.20). It was assumed that the control group would have a similar finding in the current study. It was estimated that 12 mice were required in each group (estimated with G* power, 80% power, alpha of 0.05). Sample sizes were inflated by 30%, to account for possible drop-outs due mice potentially needing to be killed due to signs of distress.

Graphpad Prism (GraphPad Software V6.0, San Diego, CA) and R studio software programs were used to analyse data. Data were tested using D’Agostino–Pearson normality test. Data with normal distribution were expressed as mean ± standard error of mean (SEM) and analysed using parametric tests. Non-normally distributed data were expressed as median and interquartile ranges (IQR) and analysed using non-parametric tests. Linear mixed effect model analyses were used to compare LDI and treadmill data between groups from the commencement of metformin or vehicle until the conclusion of the study. For these analyses, variation between individual mice was treated as random effects. LDI or treadmill data and time were treated as fixed effects. Model fit was assessed by the examination of the spread of standardized residuals and qq-normal plots. The interaction of time and treatment was used to interpret difference between groups. Data from the end of the experiment were compared between groups using a Mann–Whitney *U* test and expressed as median and IQR. For all tests, a *P* value of *<*0.05 was considered to be statistically significant.

## Results

### Metformin administration improved blood supply to ischaemic limbs

The two-stage operation created severe HLI (Figure 1B and C). Mice receiving metformin exhibited improved limb blood supply compared with the vehicle control group over the four-week monitoring period (*P*<0.001, Figure 1B and C). After four weeks of metformin administration, mean limb blood supply was approximately 15% greater than in controls (Figure 1C).

### Metformin administration did not improve physical performance

Mice receiving metformin had similar treadmill performance to mice receiving vehicle control throughout the experimental period (Figure 1D, *P*=0.225).

### Metformin administration up-regulated phosphorylation of AMPKα and eNOS in ischaemic muscles

**Figure 2:**
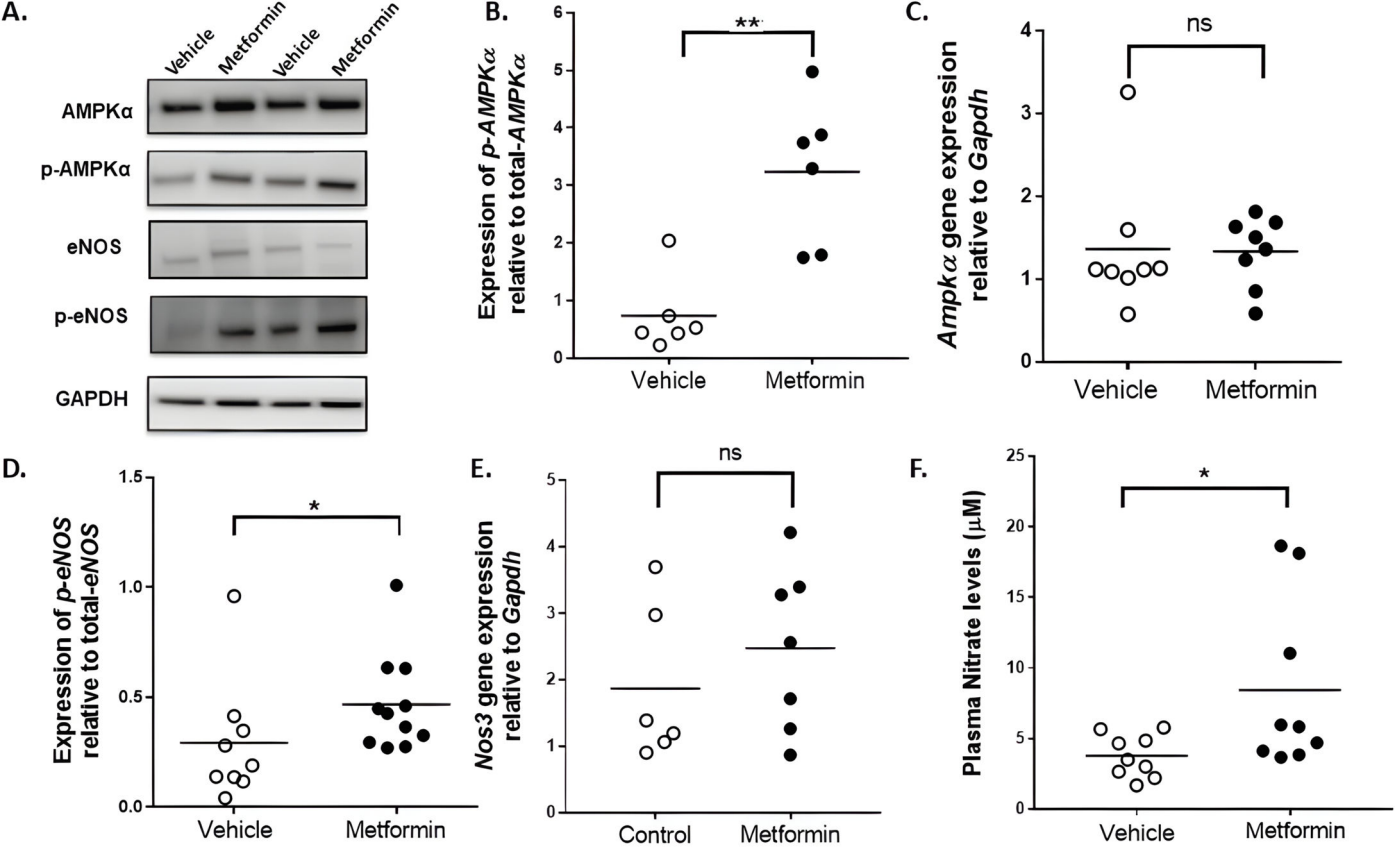
Effect of metformin administration on protein and mRNA expressions in the gastrocnemius muscle of the two-stage mouse model of PAD. **(A**) Representative immunoblots of AMPKα, phospho-AMPKα, total e-NOS, phospho-eNOS (Ser^1177^) and GAPDH expression. (**B**) Quantitative graph showing the relative densitometry analysis of phospho-AMPKα protein expression relative to total-AMPK expression. (**C**) Quantitative graph showing the *Ampkα* mRNA expression relative to the *Gapdh* expression. (**D**) Quantitative graph showing the relative densitometry analysis of phospho-eNOS protein expression relative to total-eNOS expression in the ischaemic gastrocnemius muscle. (**E**) Quantitative graph showing the *Nos3* mRNA expression relative to the *Gapdh* expression. (**F**) Effect of metformin on circulating plasma nitrate levels. Plasma levels of nitrate, four weeks after vehicle or metformin administration. Data presented as mean and individual data points and were compared between the groups using Mann–Whitney *U* test, * indicates *P<*0.05 and ‘ns’ indicates *P>*0.05. PAD, peripheral artery disease.

In mice receiving metformin, AMPKα protein expression levels were similar to controls (*P*=0.818, Figure 2A and B and Supplementary Figure S1). Mice receiving metformin had higher levels of phospho-AMPKα relative to total-AMPKα compared with vehicle controls (*P*=0.009, Figure 2A and B). The mRNA levels of *Ampkα* were similar in mice receiving metformin and vehicle (*P*=0.382, Figure 2C). Higher levels of phospho-eNOS relative to total eNOS were observed in mice receiving metformin (*P*=0.031, Figure 2D, Supplementary Figure S2 and S3). The mRNA levels of *Nos3* were similar in mice receiving metformin and vehicle (*P*=0.445, Figure 2E).

### Metformin administration increased plasma concentrations of NO

After four weeks of metformin administration, plasma NO levels were greater than controls (8.44 ± 2.02 µM and 3.77 ± 0.50 µM, *P*=0.024; Figure 2F).

### Metformin administration attenuated markers of oxidative stress and mitochondrial biogenesis in ischaemic muscles

**Figure 3:**
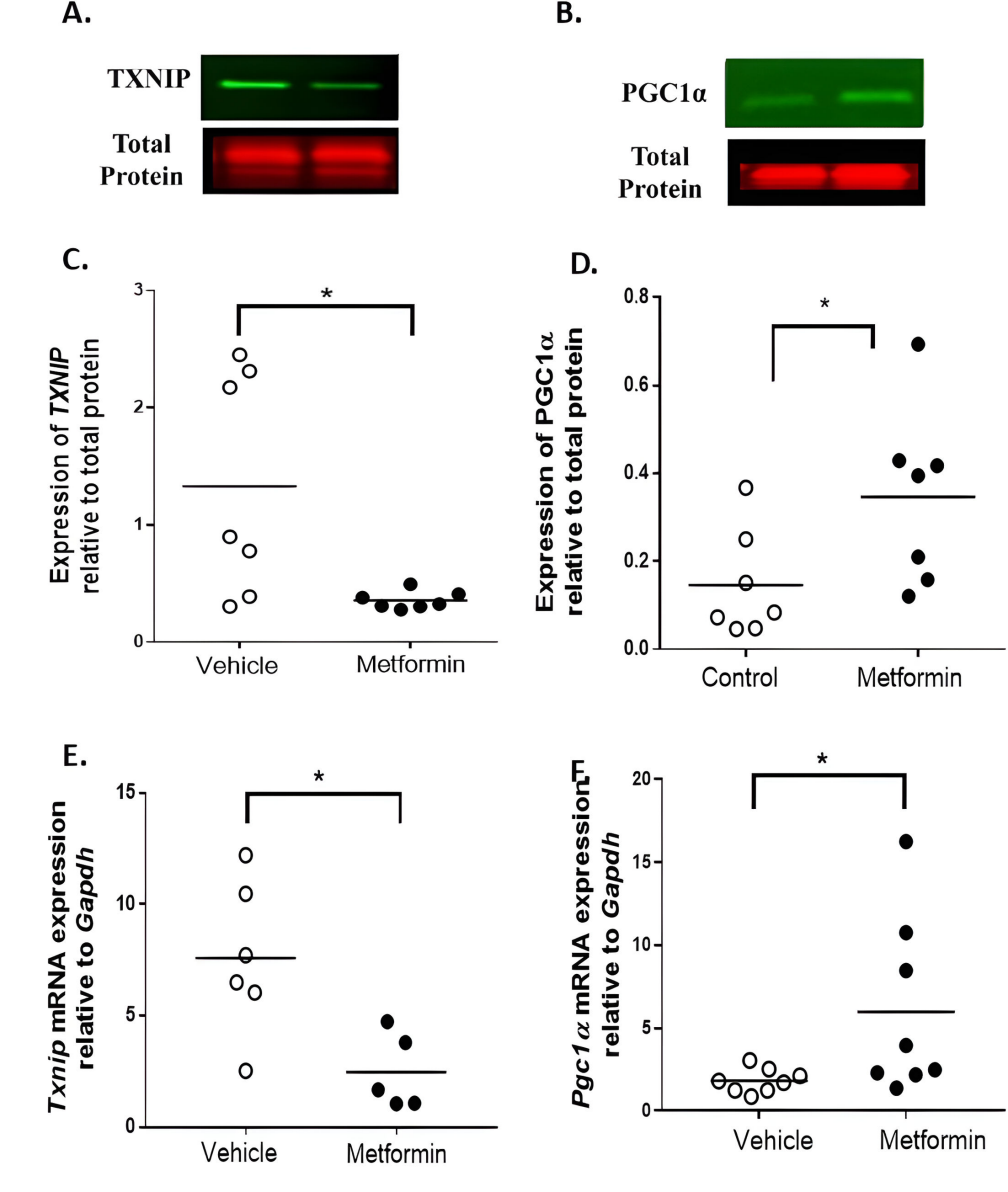
The effect of metformin on protein and mRNA expressions in the ischaemic gastrocnemius muscles of the two-stage mouse model of PAD. **(A – C**) Results of relative densitometry analysis of TXNIP protein relative to total protein expression. (**B –D**) Results of relative densitometry analysis of PGC1α protein relative to total protein expression. (**E**) Quantitative graph showing the Txnip mRNA expression relative to *Gapdh* in the ischaemic gastrocnemius muscles. (**F**) Quantitative graph showing the *Pgc1α* mRNA expression relative to *Gapdh* in the ischaemic gastrocnemius muscles. Data expressed as median and interquartile range with maximum and minimum data points (whiskers). Data were compared between groups with Mann–Whitney *U* test, * indicates *P*<0.05, *** indicates *P*<0.001. PAD, peripheral artery disease.

Relative protein and gene expression of the oxidative stress augmenting protein TXNIP was significantly down-regulated (Figure 3A, C and E, *P*=0.038 and *P*=0.017; Supplementary Figure S4) in ischaemic muscles by metformin compared with vehicle control. Metformin significantly up-regulated relative protein and gene expression of the mitochondrial biogenesis marker PGC1α (Figure 3B, D and F, *P*=0.026 and *P*=0.028; Supplementary Figure S5) in ischaemic muscles compared with controls.

## Discussion

The main finding of this study was that administering metformin for four weeks improved blood supply within a novel model of sustained limb ischaemia. This finding is in line with similar findings in the acute HLI model and two uncontrolled studies in PAD patients [17,21,22].

The two-stage HLI model has been previously shown to simulate reduced physical performance typically found in people with intermittent claudication [9]. The improvement in blood supply in response to metformin was relatively small, and there was no evidence of improvement in physical performance as assessed by a treadmill test. Assessing physical performance in mice, however, is not straightforward [27]. Ambulatory capacity improved in the control group over time, likely due to a learning effect as previously reported in clinical trials and past mouse studies [28,29]. This may have masked any benefit in ambulatory capacity because of drug treatment, and larger studies with longer follow-up are needed to more completely examine the effect of metformin. It should be noted though that it remains unclear how the improved blood supply identified in this study would translate into benefit in people with PAD, although this will become clearer with multiple clinical trials expected to report in the next few years [23].

Metformin indirectly activates AMPK by inhibiting the mitochondrial complex 1 of the electron transport chain [19]. In the current study, AMPK phosphorylation was increased by metformin and associated with eNOS phosphorylation and increased plasma NO. AMPK activation by metformin has been reported to lead to phosphorylation of eNOS resulting in increased NO bioavailability, which can promote angiogenesis [17,30]. It has been previously reported that metformin does not promote recovery of blood supply in eNOS-deficient mice in which acute HLI is induced [17]. These findings along with those from the current study suggest that eNOS is a key mediator of the action of metformin on limb reperfusion [17].

Metformin has been reported to reduce the expression of TXNIP through AMPK activation in aortic endothelial cells [18]. TXNIP has been previously reported to be a critical protein in impairing angiogenesis in mice models [31,32]. In the current study, metformin down-regulated the expression of TXNIP in ischaemic muscles. Thioredoxin is an antioxidant protein [33], and TXNIP is a negative regulator of thioredoxin [34]. TXNIP knockdown has been shown to improve blood supply in ischaemic hind limbs [32]. TXNIP knockdown is associated with increased e-NOS expression and NO production [32,35]. These findings suggest that metformin probably up-regulated NO, at least in part, by down-regulating TXNIP.

Endothelial cell migration and proliferation are key processes in angiogenesis [36]. A key regulator of this process is PGC1α, which promotes endothelial cells mitochondrial biogenesis [37,38]. Metformin has been previously reported to up-regulate PGC1α in skeletal muscles through AMPK activation [19,20]. The results of the present study suggest that metformin promotes angiogenesis, at least in part, by up-regulating PGC1α.

This study has several strengths. These include the incorporation of methods to reduce bias, such as randomisation of mice to study drug or vehicle control, the use of a pre-clinical model with pathophysiological features more comparable to human PAD than the traditional HLI model, the assessment of ambulatory capacity as a key outcome which is usually measured in human PAD clinical trials and the use of a clinically relevant drug dose. The animal model of ongoing severe ischaemia created using ameroid constrictors in older mice, as employed in this study, more closely replicates the pathophysiology of PAD patients compared with the commonly used transient ischaemia model typically established in younger mice in previous studies. Mice were randomly allocated to groups, outcomes were assessed by an investigator blinded to group allocation and sample sizes were estimated *a priori*. The study also had several limitations and caveats. Metformin did not improve the physical performance of mice, treatment time was limited to four weeks and sample sizes may have been inadequate to assess the effect of the drug on the treadmill test used. It is also possible that a higher dose of metformin may have had a more substantial effect than demonstrated in the current study. Furthermore, some outcomes, such as capillary density, were not assessed due to insufficient tissue samples being available.

In conclusion, this study suggests that metformin administration for four weeks improves blood supply in a novel mouse model of sustained limb ischaemia. This effect appeared to be driven by the activation of AMPK, up-regulation of NO and PGC1α, and down-regulation of TXNIP. Metformin is currently being investigated in clinical trials as a treatment for intermittent claudication. The results from this mouse model suggest that it is possible metformin could improve limb blood supply without improving walking performance.

**Clinical Perspectives**

- There are few drug therapies for walking impairment caused by peripheral artery disease.
- In a clinically relevant mouse model of peripheral artery disease, metformin improved hind limb blood supply but not ambulation.
- The findings suggest that laser Doppler hind limb blood supply may not be an ideal outcome measure to gauge the success of a drug in patients with peripheral artery disease-related walking impairment.

## Supplementary Material

Online supplementary material

## Data Availability

The data from this article is contained within the paper and there are no mandatory deposited data included.

## Abbreviations

AMPK: 5′ adenosine monophosphate-activated protein kinase
HLI: hind limb ischaemia
IQR: interquartile range
LDI: laser Doppler imaging
OCT: optimal cutting compound
PAD: peripheral artery disease
PGC1α: peroxisome proliferator-activated receptor gamma coactivator 1-α
PU: perfusion units
TXNIP: thioredoxin interacting protein
eNOS: endothelial nitric oxide synthase
