# Supplementary material for: Effect of metformin in a novel experimental model of peripheral artery disease
Source: Clin Sci (Lond). 2025 Aug 13;139(15):847–56. doi: 10.1042/CS20243343 (PMC12493163; doi:10.1042/CS20243343)

***Supplementary Tables***

**Supplementary Table S1. Table listing antibodies used for protein expression assays by Western blotting**

| **Antibody** | | **Species** | **Size of band detected (kDa)** | **Dilution in (TBS + 0.05% Tween)** | **Supplier & Catalogue** |
| --- | --- | --- | --- | --- | --- |
| **Primary** | GAPDH | Rabbit | 37 | 1:10,000 | Cell signalling Technology #5174 |
|  | TXNIP | Rabbit | 60 | 1:1000 | Cell signalling Technology #14715 |
|  | PGC1α | Rabbit | 92 | 1:1000 | Abcam #ab54481 |
|  | AMPKα | Rabbit | 62 | 1:1000 | Cell signalling Technology #5832 |
|  | Phospho- AMPKα (Thr 172) | Rabbit | 62 | 1:1000 | Cell signalling Technology #2535 |
|  | e-NOS | Rabbit | 140 | 1:1000 | Cell signalling Technology #9572 |
|  | Phospho-eNOS (Ser1177) | Rabbit | 140 | 1:1000 | Cell signalling Technology #9571 |
| **Secondary** | Anti- rabbit IgG HRP conjugated | Goat |  | 1:1000 | abcam #ab6721 |
|  | Fluorophore conjugated anti-rabbit IgG­­­­­­­­ (IRDye 800CW anti-rabbit IgG) | Donkey |  | 1:15,000 | Li-Cor #925-32213  (Millenium Sciences) |

**Supplementary Table S2. Table listing primers used for mRNA expression assays by qRT-PCR**

| **Gene Name** | **Gene** | **Species** | **Quantitect primer assay** | **Supplier** |
| --- | --- | --- | --- | --- |
| AMP-activated protein kinase | *Ampk* | Mouse | QT00286923 | Qiagen |
| Thioredoxin Interacting Protein | *Txnip* | Mouse | QT00296513 | Qiagen |
| Peroxisome proliferator-activated receptor gamma coactivator 1 | *Pgc1* | Mouse | QT02524242 | Qiagen |
| Nitric oxide synthase 3 | *Nos3* | Mouse | QT00152754 | Qiagen |
| Glyceraldehyde 3-phosphate dehydrogenase | *Gapdh* | Mouse | QT01658692 | Qiagen |

**Supplementary Figures**

**Figure S1.** Western blot images of AMPKα (A) and phospho-AMPKα expression (B) with their respective GAPDH blots for the effect of metformin on limb ischemia. Unlabelled lanes contained ladders. Red box shows the bands used as representative bands in figure 2.

**A. B.**


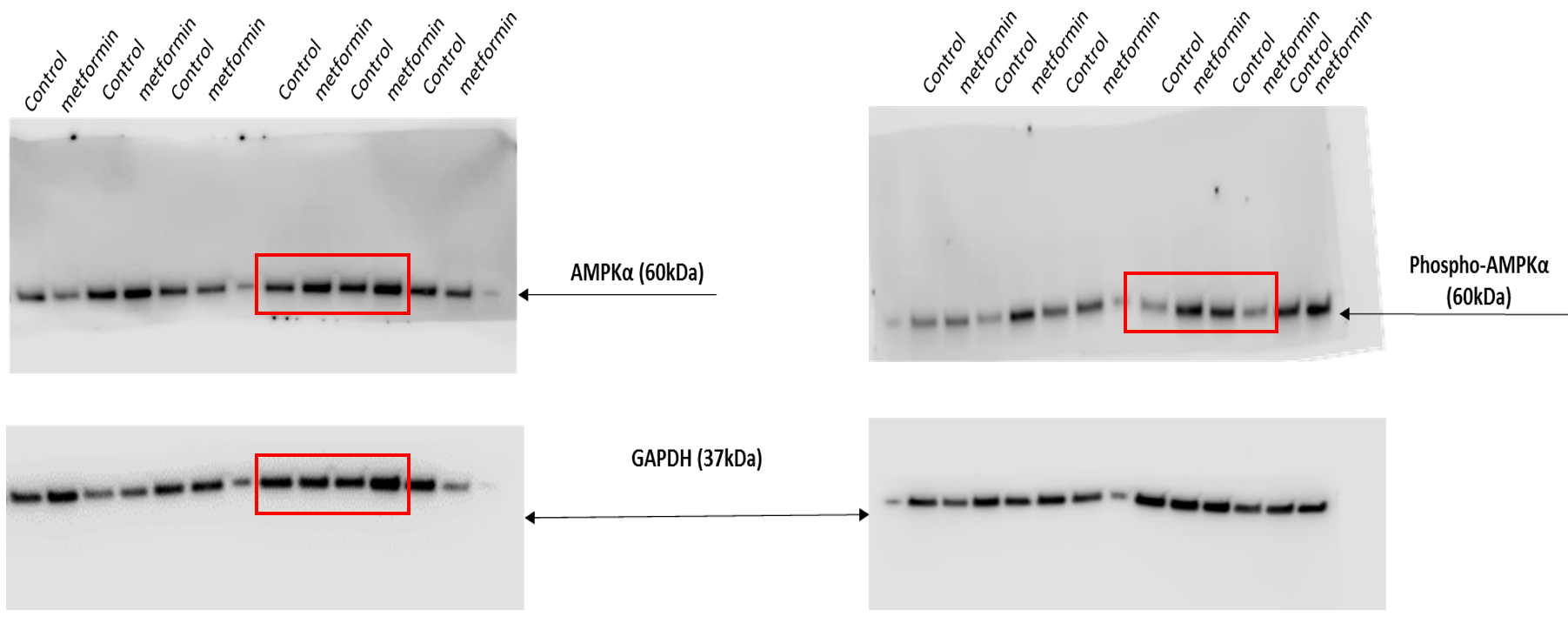


**Figure S2. Western blot images of e-NOS expression with their respective GAPDH blots.** Unlabelled lanes contained ladders. Red box shows the bands used as representative bands in figure 2.


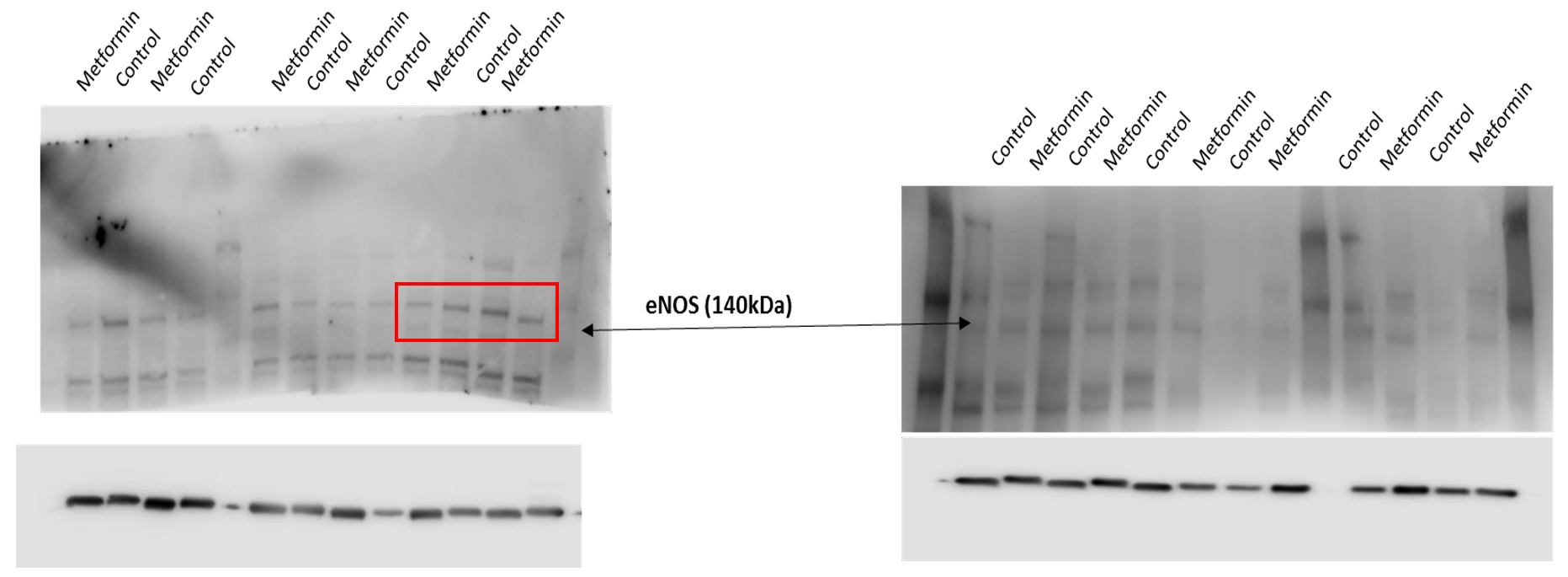


**Figure S3.** Western blot images of phospho-eNOS expression with their respective GAPDH blots. Red arrows indicate samples excluded due to signal intensity being not quantifiable. Unlabelled lanes are contained ladders. Red box shows the bands used as representative bands in figure 2.


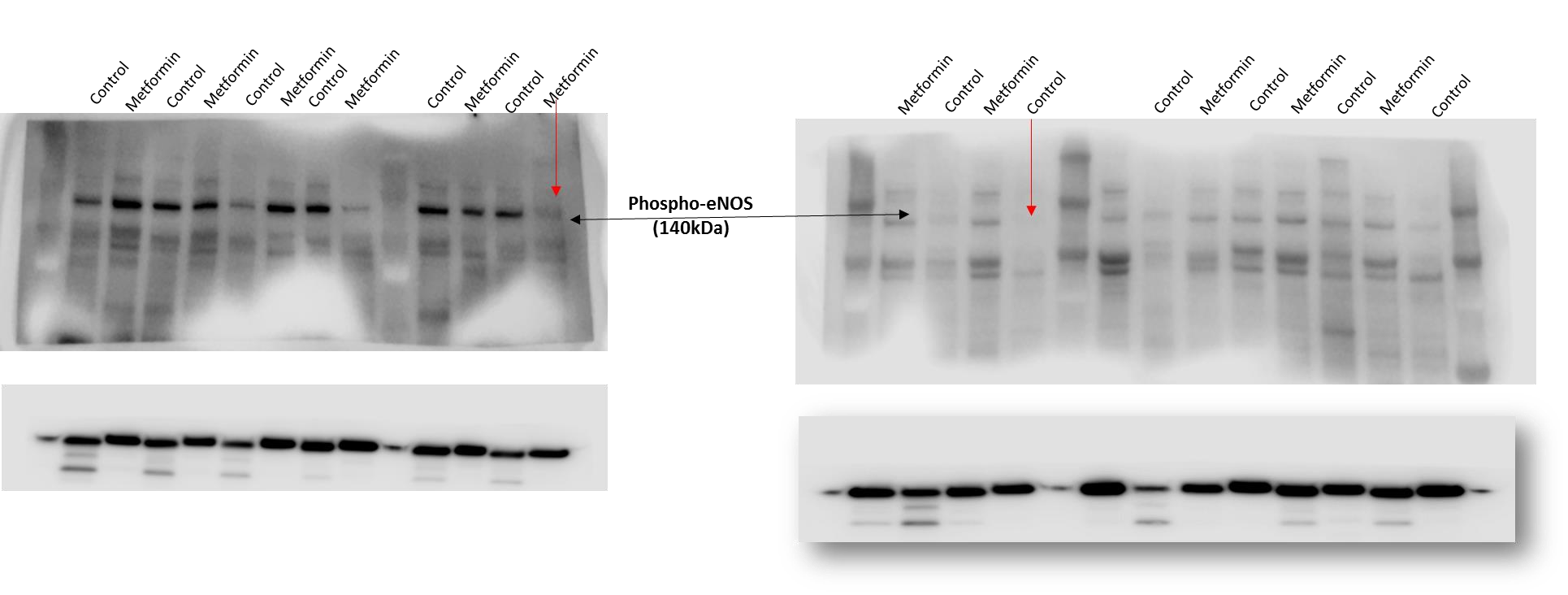


**
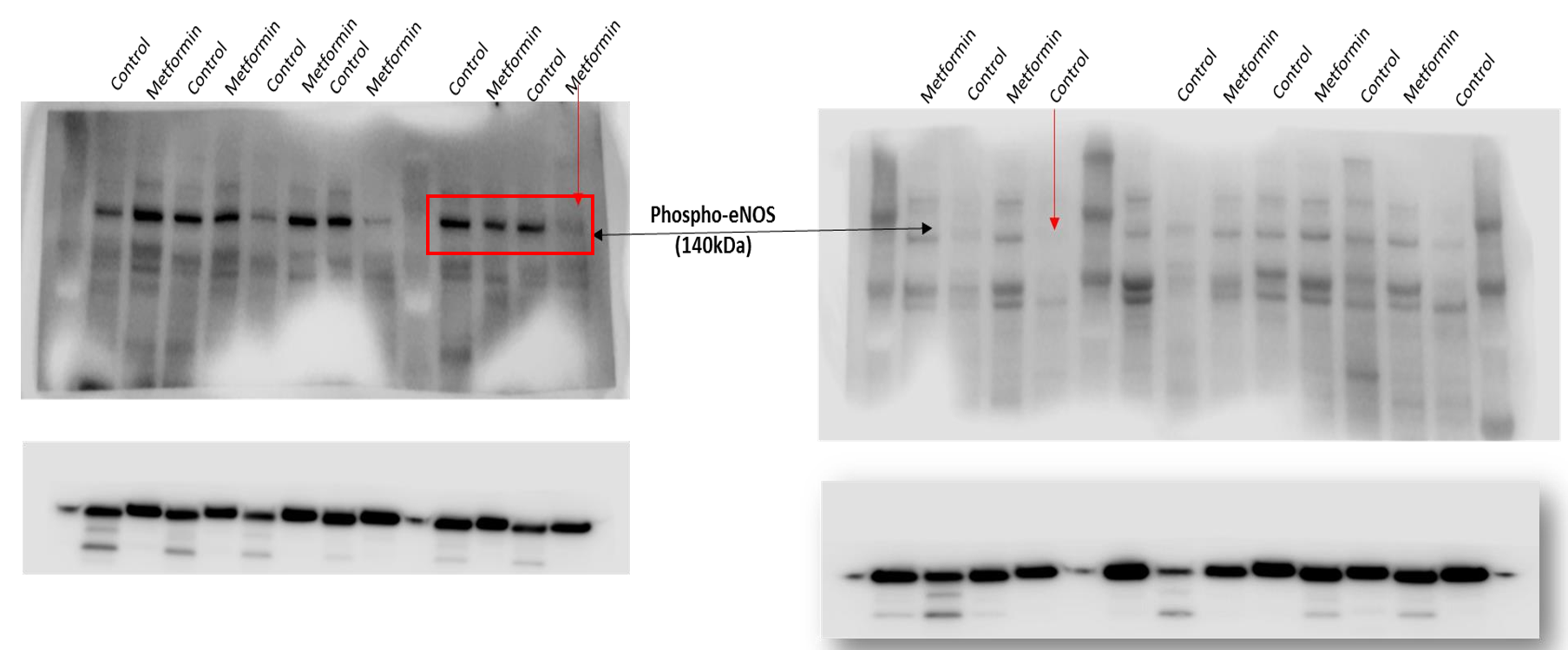
**

**Figure S4.** Western blot images of TXNIP expression with their respective total protein expression blots. Red labels indicate samples excluded due to artefacts in lane interfering with quantitation of signal intensity. Yellow box shows the bands used as representative bands in figure 3.


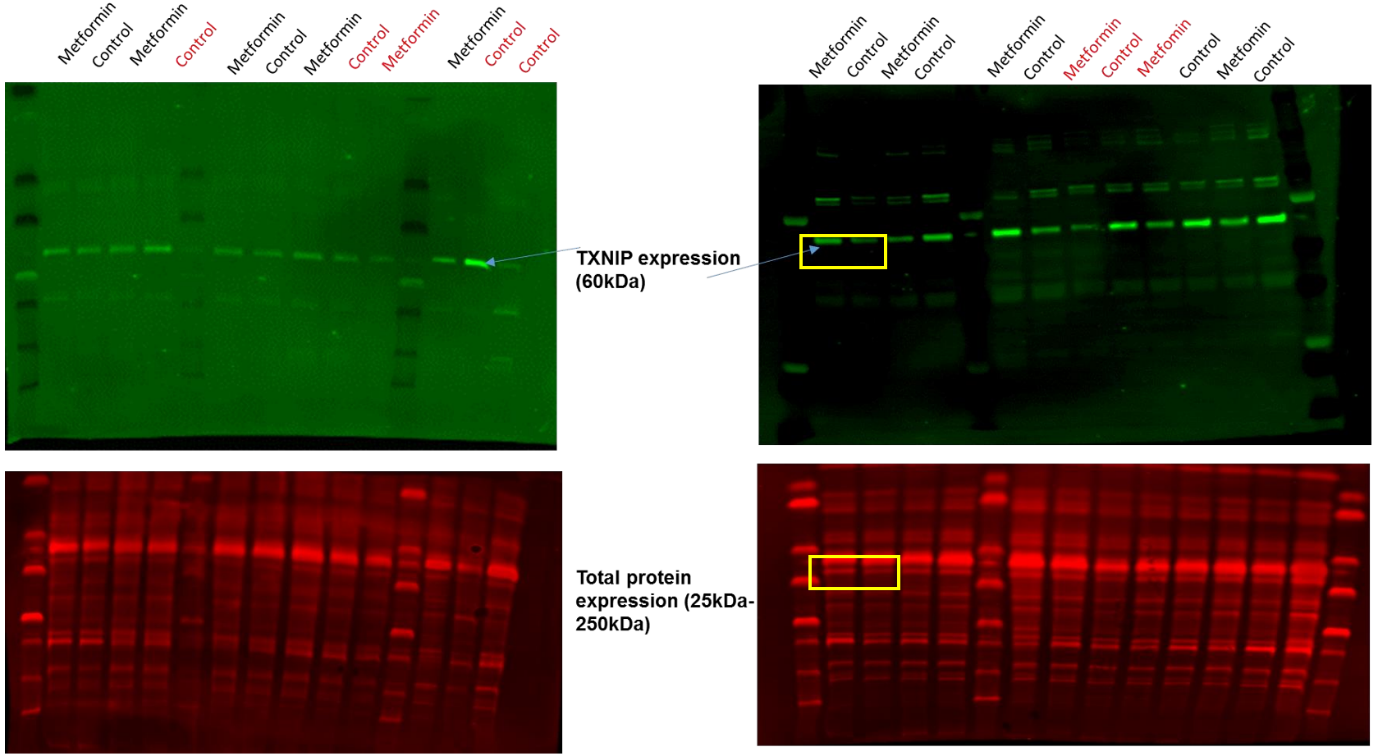


**Figure S5.** Western blot images of PGC1α expression with their respective total protein expression blots. Red labels indicate samples excluded due to artefacts in lane interfering with quantitation of signal intensity. Yellow box shows the bands used as representative bands in figure 3.


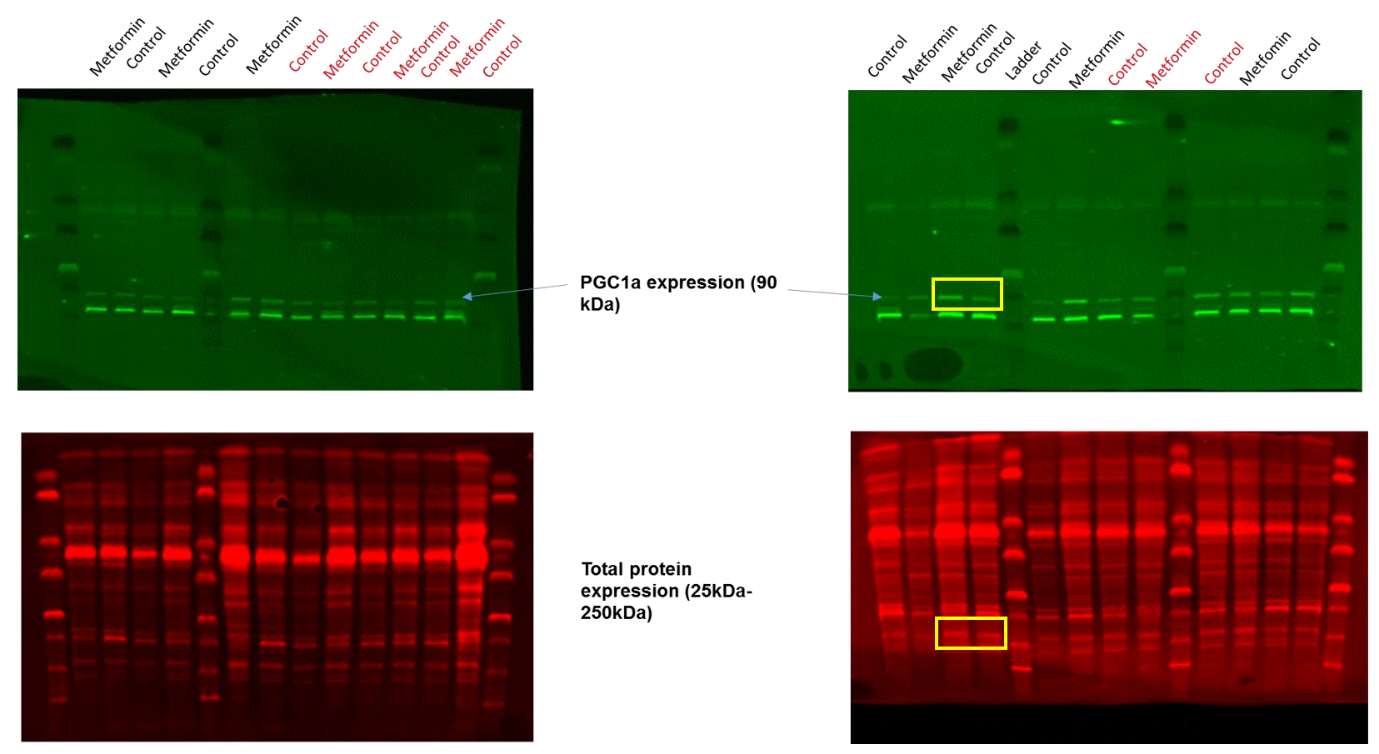

Supplement: Online supplementary material [file cs-139-15-CS20243343-s001.docx]
